# Transcatheter aortic valve implantation under 75 years of age: only for high surgical risk patients; but for how long?

**DOI:** 10.1007/s12471-024-01900-9

**Published:** 2024-08-29

**Authors:** Michiel Voskuil, Michael G. Dickinson

**Affiliations:** Department of Cardiology, University Medical Center Utrecht, Utrecht, The Netherlands

Transcatheter aortic valve implantation (TAVI) has progressively emerged as a valid treatment option in prohibitive, high, intermediate and, more recently, even in low surgical risk patients as assessed by the Society of Thoracic Surgeons (STS) score [1]. In 2020, the Netherlands Society of Cardiology (NVVC) and the Netherlands Society for Thoracic Surgery (NVT) jointly published the Indication Guideline to facilitate the heart team with major and minor criteria for the selection of patients most suitable for TAVI [2]. One important aspect in this document is age. According to the document, only patients aged > 80 years with the presence of additional risk factors are eligible for TAVI. Previously, it was shown that the outcome of TAVI in patients > 85 years in the Netherlands is comparable with that of those aged ≤ 85 years [3]. However, also younger patients < 80 years are regularly treated with TAVI in clinical practice. According to the guidelines for the management of valvular heart disease of the European Society of Cardiology (ESC), TAVI is even recommended for patients from ≥ 75 years, for patients at high risk (STS score/EuroSCORE II > 8%) and for those unsuitable for surgery [4]. The 2020 American guidelines are even more liberal, stating that TAVI should be considered for patients from 65 to 80 years of age using shared decision-making based on a ‘balance between expected patient longevity and valve durability’ [5].

In this issue of the Netherlands Heart Journal, Adrichem et al. report on their retrospective analysis of all consecutive patients aged 50–75 years who underwent surgical aortic valve replacement (SAVR) or TAVI at their centre [6]. The aim of the study was to define the phenotypes that may clarify why patients of this relatively young age would undergo TAVI instead of SAVR and, second, report on the clinical outcome of this patient population. A total of 678 patients were included (292 TAVI and 386 SAVR patients) with a median age of 69 years (64–72), of whom 439 patients (64.7%) were male. The median EuroSCORE II was 1.8% (1.1–3.2%). TAVI patients were slightly older (70 vs 68 years in the SAVR group). Furthermore, more TAVI patients had a reduced left ventricular ejection fraction (LVEF < 50%; 31.8% vs 18.7%), diabetes (40.8% vs 26.4%), atrial fibrillation (26.4% vs 14.2%) and chronic kidney disease (29.1% vs 12.7%). Assessment of the distribution of risk criteria showed 6 of the 8 high-risk criteria and 8 of the 9 very high-risk criteria were significantly more prevalent in the TAVI group, respectively. Also, within the different risk cohorts, TAVI and SAVR patients differed significantly regarding non-cardiovascular comorbidities, peripheral artery disease and frailty. Nevertheless, there was no difference in 30-day all-cause mortality between TAVI and SAVR patients, although mortality was higher for TAVI than for SAVR patients at 1 year, as well as at 5‑year follow-up. This difference was mainly driven by the presence of an active malignancy, liver cirrhosis or the use of immunomodulatory drugs, which was associated with a 4.5 times higher risk for 5‑year mortality. They conclude that in patients aged < 75 years there is a distinct difference in risk profile between those undergoing TAVI and SAVR. Second, the higher 1‑year and 5‑year mortality rate in the TAVI patients may be explained by the overall higher prevalence of high-risk characteristics in the TAVI group. Finally, they conclude that application of the Dutch risk criteria was only partially effective in determining a patient’s actual risk.

The results from the study by Adrichem et al. are in line with other studies such as the subanalysis from the European SOURCE 3 Registry, which showed that 12% of patients undergoing TAVI were < 75 years [7]. They also conclude that these younger patients had a higher incidence of comorbidities, particularly those not included in traditional surgical risk score assessment tools, such as severe liver disease, previous radiation therapy and porcelain aorta. That is the reason that in the Dutch Indication Guideline from 2020 many of these comorbidities were included in the document. Most likely, the clinical judgement by the local heart teams with these agreed parameters in hand is more accurate in making the right decision for this patient cohort, compared with the traditional risk scores alone [8].

Finally, as also stated above, several recent trials have shown non-inferiority or even superiority of TAVI over SAVR in patients with symptomatic aortic valve stenosis with low surgical risk who are in general younger [1]. Therefore we can safely state that TAVI is evolving from being a valid treatment option for young patients with a high surgical risk to a larger group of relatively young patients with aortic valve stenosis being eligible for this minimal invasive treatment option. Future studies need to focus more on the determination of the exact threshold of age and comorbidities for this patient group that will benefit from TAVI.
